# Dr. William a Hammond

**Published:** 1900-02

**Authors:** 


DR. WILLIAM A HAMMOND, of Washington, D. C., Surgeon-General U. S. Army (retired), died at his home, January 6, 1900, aged 71 years. Dr. Hammond had a stormy career, especially during the Civil War period when he was Surgeon-General of the Army. He was dismissed from the service for alleged irregularities in medical contracts for supplies, but some years afterward he was reinstated after complete review of the case, when he was placed on the retired list. He made fame as an author and as a neurologist, rising from almost abject poverty after the war to wealth within the next twenty-five years. He subsequently became somewhat embarrassed, it is reported, but finally extracted himself, and lived a life of comparative ease in his later years. He was buried with military honors at Arlington National Cemetery.

